# Herbal medication triggering lupus nephritis - a case report

**DOI:** 10.1186/s12906-020-02971-y

**Published:** 2020-06-02

**Authors:** Anna Misya’il Abdul Rashid, Fauzah Abd Ghani, Liyana Najwa Inche Mat, Christopher Thiam Seong Lim

**Affiliations:** 1 Department of Medicine, Faculty of Medicine and Health Sciences, Universiti Putra Malaysia, Serdang, Selangor 43400 Malaysia; 2 Department of Pathology, Faculty of Medicine and Health Sciences, Universiti Putra Malaysia, Serdang, Selangor 43400 Malaysia

**Keywords:** Lupus nephritis, Drug-induced lupus, Herbal supplement

## Abstract

**Background:**

Herbal medication is widely used in our region as a mode of alternative medicine. Its contents and combinations are often modified to suit the needs of different populations. These products are said to boost the immune system and may serve as a protective measure against many diseases including Systemic Lupus Erythematosus (SLE). Some even lay claims to be able to cure SLE. Although they are not without side effects, these medications are still preferred due to their widespread availability and affordability, compared to modern medications. However, to date, there have been no reported cases in which these traditional medications can trigger a lupus-like reaction, moreover one involving the kidneys.

**Case presentation:**

We report a patient who developed overt lupus nephritis after consuming a course of herbal supplement. Her renal status did not improve upon cessation of the offending drug, and she required immunosuppressive therapy. After one cycle of IV cyclophosphamide, we managed to get the patient into remission - she is now on tapering doses of steroids.

**Conclusion:**

We wish to highlight the possibility of consumption of herbal medication and the emergence of drug-induced lupus nephritis. A thorough anamnesis and high index of suspicion of drug-induced lupus nephritis is warranted when a patient on supplements presents with urinary abnormalities.

## Background

Drug-induced lupus (DIL) is a rare adverse reaction to a variety of drugs. Over 80 drugs have been implicated in DIL - hydralazine, procainamide, quinidine, and minocycline being few of the most well described triggers [1]. Patients typically present as early as 1–2 months after the drug exposure, with fever, weight loss and fatigue, along with musculoskeletal complaints, most frequently arthralgia [2]. Rarely the kidneys are involved, although there have been isolated case reports describing occurrence of renal lupus-like syndrome after exposure to penicillamine [3] and propylthiouracil [4]. Herbal medications are also known to cause acute kidney injuries, [5, 6] however there are no cases yet reported to trigger a plants a lupus-like syndrome involving the renal tissue.

## Case report

**Fig. 1:**
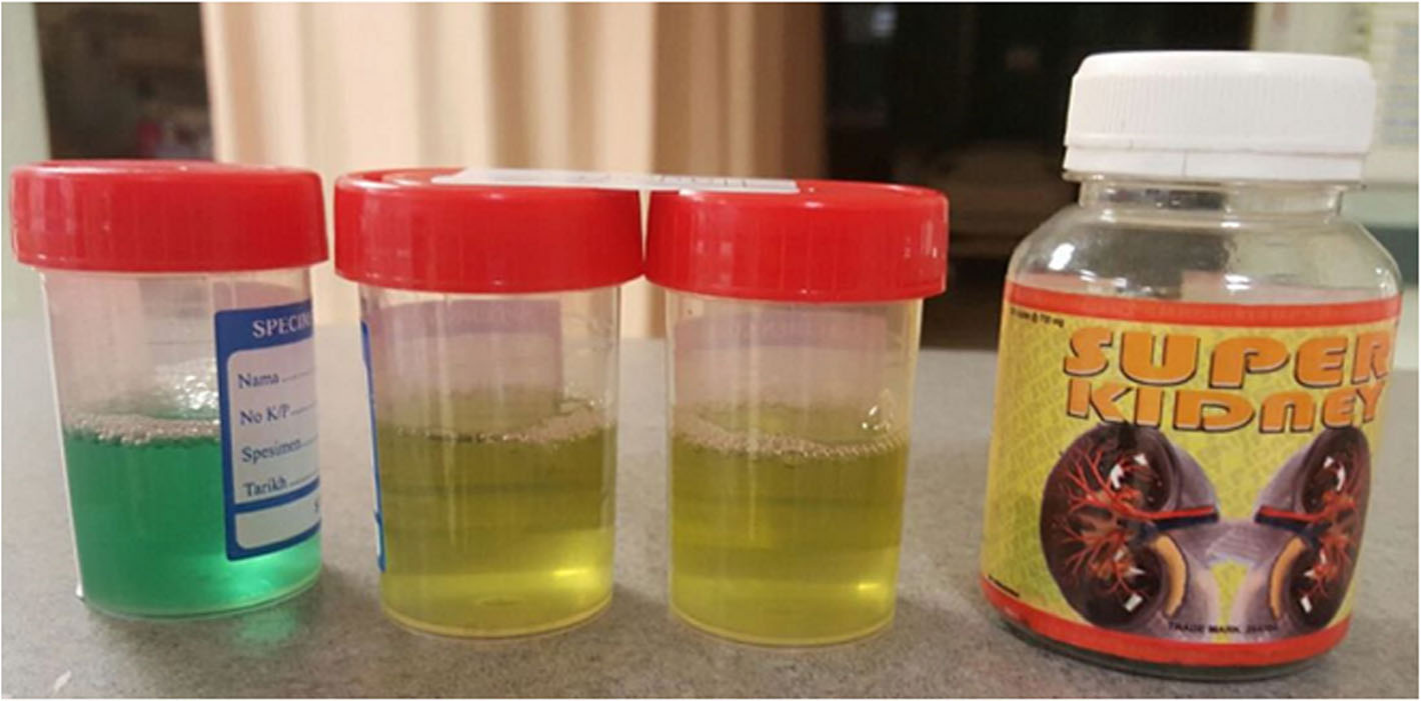
Green discoloration of urine (left) along with the offending drug (right)

**Fig. 2:**
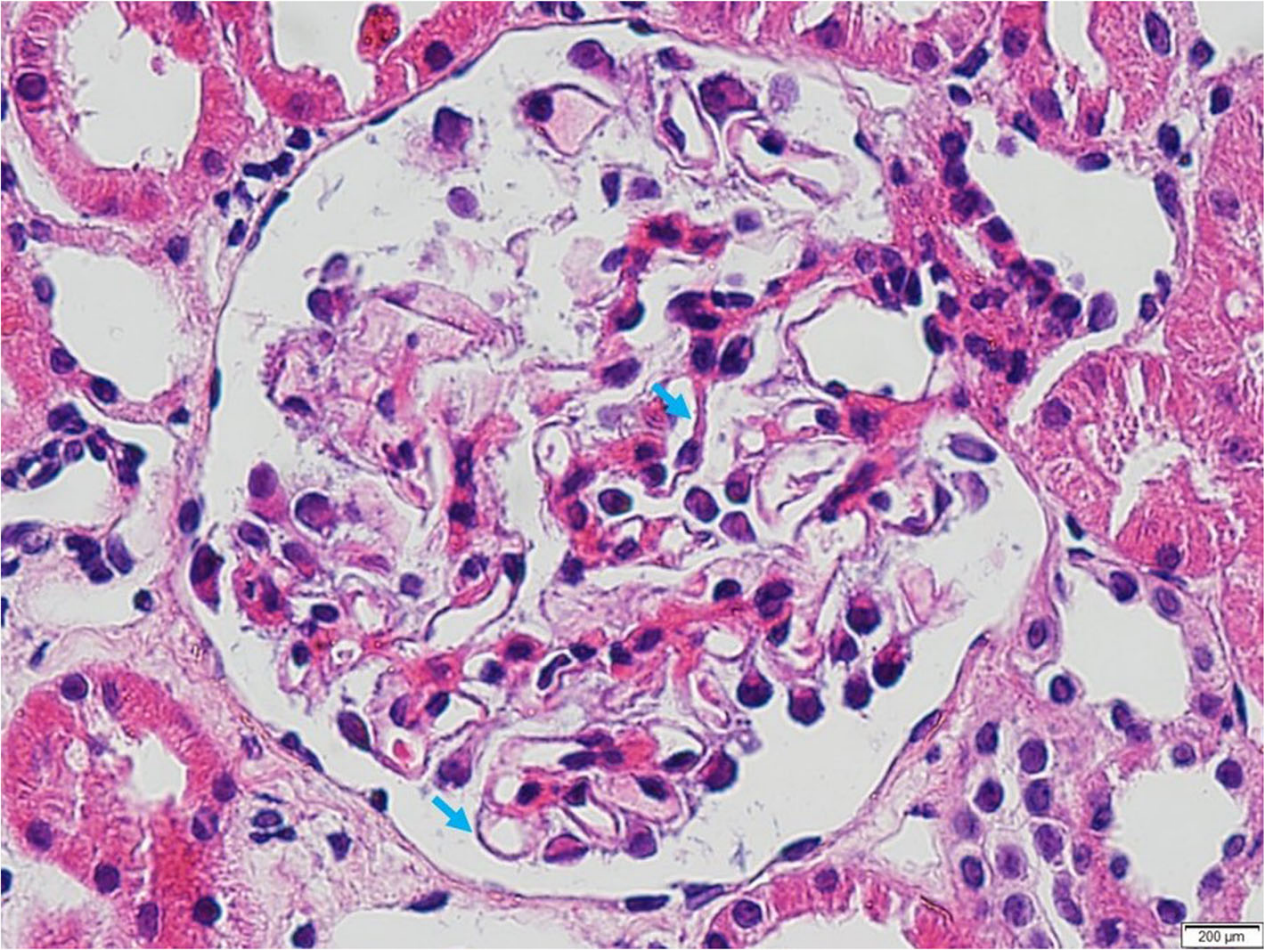
Rigid and thickened glomerular basement membrane (Haematoxylin & Eosin (H&E), original magnification × 60)

**Fig. 3:**
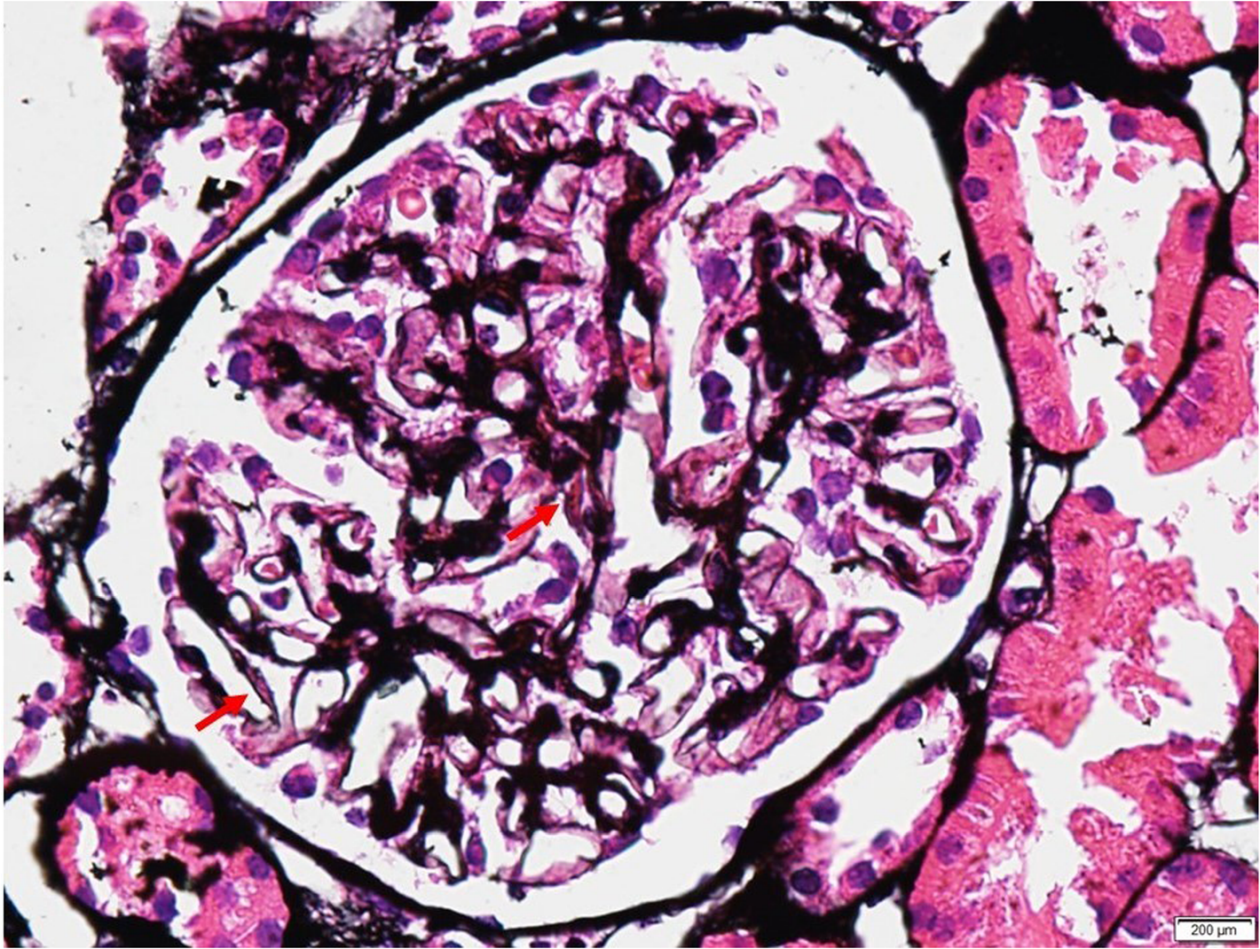
Tram track along glomerular basement membrane (Periodic acid-silver methenamine (PAAG) silver stain, original magnification × 60)

**Fig. 4:**
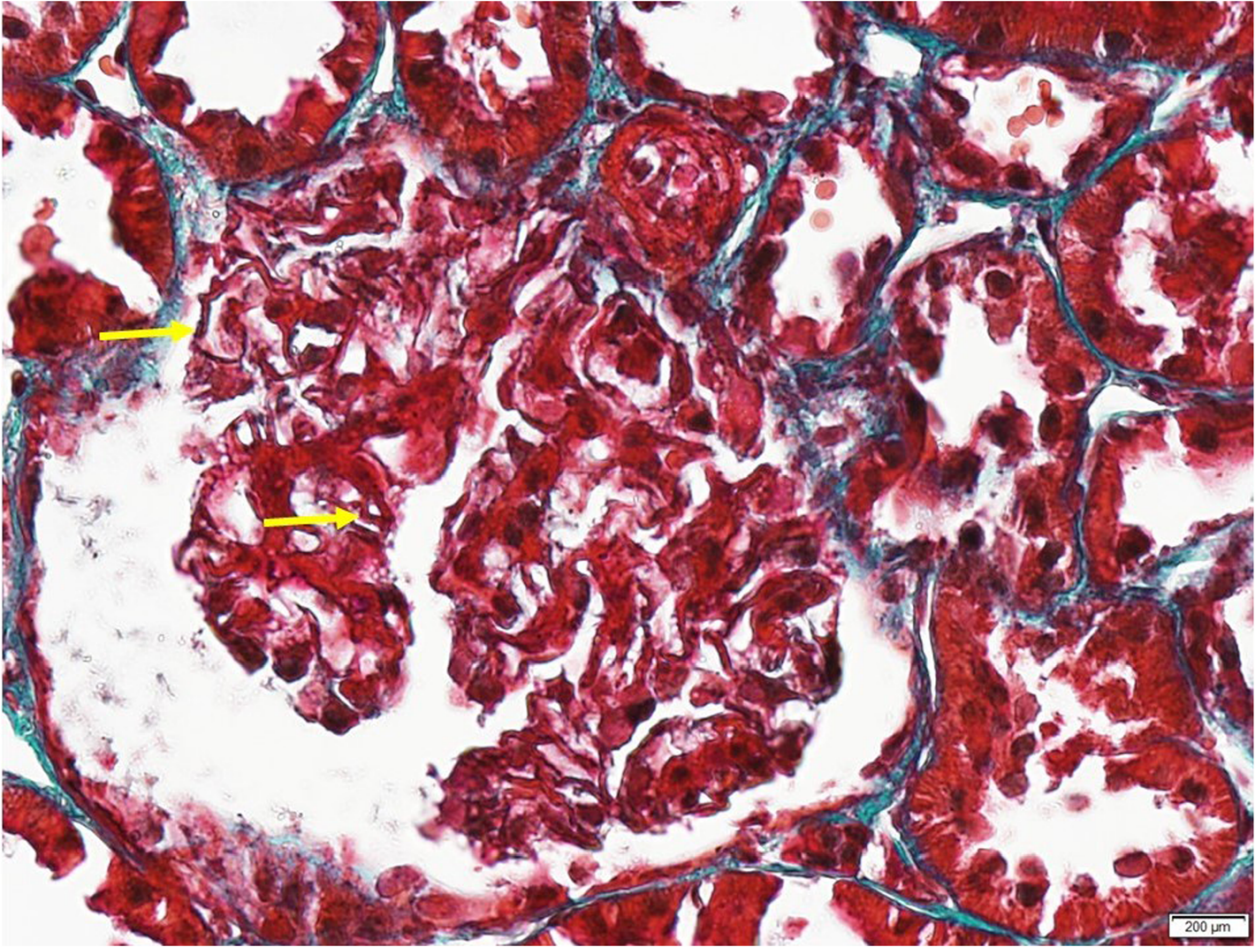
Subendothelial deposition with splitting along rigid capillary loops (Masson Trichrome (MT), original magnification × 60)

**Fig. 5:**
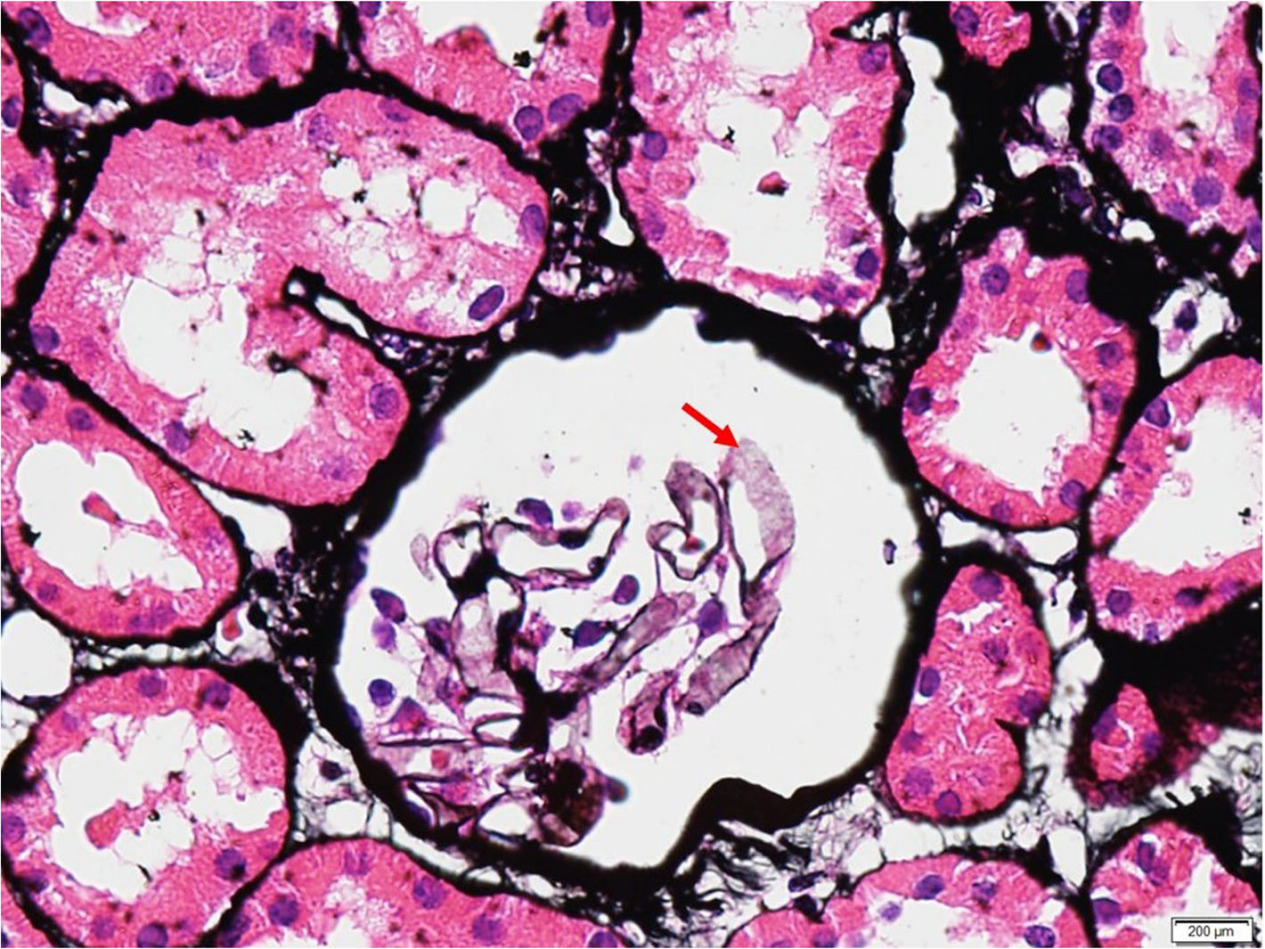
Vacuolation along glomerular basement membrane (Periodic acid-silver methenamine (PAAG) silver stain, original magnification × 60)

We report a case of a 29-year-old female who presented to us in August 2015, presenting with green discoloration and frothy urine associated with lower limb edema (Fig. 1). These symptoms were not preceded by any infective episodes. She was previously well and was only admitted for previous childbirth of which all her blood investigations were normal in 2014. In addition, she did not have any extra-renal symptoms, such as arthritis, serositis, cutaneous, or hematologic involvement. She was not on any medications but admits to using an herbal supplement named ‘Super Kidney’ for the past 6 months, containing ginseng, plantaginis folium, orthosiphonis, strobilanthi folium and retrofracti fructus, which are plants used traditionally for improving general well-being and diuresis. The supplement was not registered with the National Pharmaceutical Regulatory Agency (NPRA), thus its safety profile and detailed content was not available. On further questioning, the patient admitted that the supplement was brought from overseas. During this visit however, urine dipstick revealed 4 + proteinuria and 24 h urine protein was 10 g. Her creatinine was normal at 47 μmol/L, albumin was low at 11 g/L and her peripheral blood counts were normal. Further investigations revealed a negative hepatitis B, C, and HIV serologies, ANA positive with 1:640 titer, C3 and C4 levels were low at 0.78 g/L and 0.14 g/L respectively, anti-smith antibody, anti-RNP antibody, anti-Jo antibody and anti-Scl 70 antibody were negative. However anti-SSA (anti Ro) antibody and anti-SSB (anti La) antibody were positive. We were not able to send anti-histone antibody due to the non-availability of reagent at that time. Unfortunately, we did not send the serum PLA2R antibodies. Her erythrocyte sedimentation rate was high 120 mm/hr. but her C-reactive protein was normal 1.86 mg/dl. The renal biopsy showed diffuse membranoproliferative pattern composed of rigid and thickened capillary walls (Fig. 2) with presence of subendothelial depositions (Fig. 3) and splitting of glomerular basement membrane in Masson Trichrome (MT) (Fig. 4). Focal subepithelial vacuolation (Fig. 5) and focal regions of mesangial hypercellularity were observed. Immunofluorescence studies showed granular capillary loop and milder degree of mesangial immunopositivity for IgG (3+), IgA (3+), C3 (2+), C1q (3+), Kappa (1+) and Lambda (1+). Additional C4d immunohistochemistry showed granular positivity along the capillary walls. At this juncture, lupus nephritis ISN/RPS (2003) of Class IV-S (A/C) and V, and secondary membranoproliferative glomerulonephritis were considered. Putting the renal biopsy, laboratory parameters and clinical presentation into perspective, it is conceivable that this was likely an autoimmune lupus-like reaction triggered by the supplements that she had been taking. We withdrew the herbal medications and treated her with a course of 1 mg/kg prednisolone daily and low molecular weight heparin injection. Six weeks later, her edema significantly reduced, and her urine regained its normal colour. Her urine dipstick was still 4+, but her proteinuria improved to 3.8 g/24 h. Serum creatinine remained normal at 62 μmol/L and her albumin level improved to 15 g/L. Hydroxychloroquine and angiotensin converting enzyme inhibitors (ACEi) were added. Despite 9 weeks of intensive treatment, she was persistently proteinuric with serum creatinine 66 μmol/L, 24-h urine protein of 5.6 g and albumin 17 g/L. We then decided to start her on IV cyclophosphamide induction. She tolerated 6 courses of 5.5 g in the period of 6 months and had no undesirable side effects. Her response was favorable; she achieved remission after the 6th dose. We continued her on oral prednisolone and managed to taper it to a maintenance dose of 5 mg per day.

## Discussion and conclusion

Herbal medicine is a form of treatment based on the use of plants or plant extracts that may be eaten or applied to the skin to improve general well-being. Herbal medicine has been used by many different cultures throughout the world to treat illnesses, and to assist various bodily functions including SLE.

Although DIL is well described in literature, there is still no uniform criteria to establish its diagnosis. Diagnosis is primarily made based on the temporal association with drug administration and improvement of symptoms upon withdrawal of the offending drug. In most cases of DIL, symptoms are similar to SLE, but much less severe. Renal and central nervous system involvement in cases of DIL is very rare. Laboratory testing may detect the presence of auto antibodies but its presence is not a prerequisite for the diagnosis of DIL [7].

Herbal medications are known to cause acute kidney injury by virtue of naturally occurring acids that are nephrotoxic. The anatomy of the kidneys, with large endothelial surface allows toxins to be filtered through easily; making it vulnerable to toxic injuries [6]. The types of injuries may be diverse, ranging from acute tubular necrosis, acute interstitial nephritis, fibrosis, chronic interstitial nephritis, malignancies, and several types of electrolytes disorders [5, 6]. It is difficult to pinpoint the exact content or substrate that induces these changes owing to the fact that there are thousands of naturally occurring plants used for medicinal value worldwide. Some of these herbs are also used in combination with other herbs, making identification almost impossible. Furthermore, as they are marketed as health supplements, there was no strict criteria for surveillance of safety profile by the health authorities, making identification of manufacturers and distributors almost impossible.

In our case, the temporal association between the onset of symptoms and the use of the herbal medication was definite. The patient previously had no abnormalities in the laboratory investigations and was asymptomatic; until the introduction of the offending supplement. The use of the supplement may have triggered a nephrotic syndrome that when further investigated, revealed an autoimmune etiology; as evidenced by positive antinuclear antibody with low levels of serum complement. Moreover, she did not develop extra-renal symptoms, such as arthritis, serositis, cutaneous, or hematologic involvement. This by itself does not fulfill the usual Systemic Lupus International Collaborating Clinics Classification Criteria for Systemic Lupus Erythematosus (SLICC) [8] criteria for the diagnosis of SLE, however the positive renal biopsy showing evidence of class V lupus nephritis (LN) is sufficient. The mechanism of autoimmunity is complex, but is hypothesized to be due to the capability of certain lupus inducing drugs or their metabolites to form stable complexes and directly stimulates lymphocytes, causing autoantibodies to develop as an immune response [2]. Some studies also suggest a genetic predisposition in slow acetylators with HLA-DR2, HLADR3 and HLA-DR4 haplotypes being more likely to be affected with DIL [7].

Most cases of DIL will resolve within weeks within stoppage of the offending drug. No treatment is generally needed. We postulate that there might be a few explanations to the patient’s renal status not improving despite discontinuation of the offending drug. Firstly, this may reflect an atypical presentation of DIL in which the renal function did not improve after stopping the drug, and secondly, the possibility of this patient having SLE, by chance, during the period of supplement ingestion or discontinuation cannot be totally excluded. This patient’s DIL of class V LN only responded to the initiation of cyclophosphamide as per American College of Rheumatology Guideline (ACR) guidelines [9]. Whether this is a true DIL or the herbal medication triggering a preexisting lupus in our patient, remains uncertain.

This case highlights the possibility of herbal medication exposing drug-induced lupus nephritis. We wish to highlight the atypical presentation course of our DIL nephritis whereby it mimics the natural course of idiopathic lupus nephritis and needed dual immunosuppressive therapy for 6 months before achieving resolution. A thorough anamnesis and high index of suspicion of DIL is warranted when a patient on supplements presents with urinary abnormalities. It is also important to note the health implications of herbal medicines, warranting a stricter governance of these manufacturers and distributors by local health authorities.

## Data Availability

All data generated or analysed during this study are included in this published article.

## Abbreviations

DIL: Drug-induced lupus
SLE: Systemic Lupus Erythematosus
SLICC: Systemic Lupus International Collaborating Clinics Classification Criteria for Systemic Lupus Erythematosus
LN: lupus nephritis
ACR: American College of Rheumatology Guideline
